# Evaluation of Reablement Home Care: Effects on Care Attendants, Care Recipients, and Family Caregivers

**DOI:** 10.3390/ijerph17238784

**Published:** 2020-11-26

**Authors:** Yu-Hsien Chiang, Hui-Chuan Hsu, Chiung-Ling Chen, Chen-Fen Chen, Shu-Nu Chang-Lee, Ya-Mei Chen, Shang-Wei Hsu

**Affiliations:** 1 Department of Gerontechnology and Service Management, Nan Kai University of Technology, Nantou 542021, Taiwan; dada1210@redcross.tw; 2 Research Center of Health Equity, School of Public Health, College of Public Health, Taipei Medical University, Taipei 11031, Taiwan; 3 Department of Occupational Therapy, Chung Shan Medical University, Taichung 40201, Taiwan; joelin@csmu.edu.tw; 4 Department of Social Welfare, Chinese Culture University, Taipei 11114, Taiwan; czf2@faculty.pccu.edu.tw; 5 Department of Long-Term Care, National Quemoy University, Kinmen 89250, Taiwan; shunu@nqu.edu.tw; 6 Institute of Health Policy and Management, National Taiwan University, Taipei 10617, Taiwan; chenyamei@ntu.edu.tw; 7 National Defense of Medical Center, School of Public Health, Taipei 11490, Taiwan; shangweihsu@gmail.com

**Keywords:** home care, care assistants, family caregivers, older adults, reablement, long-term care

## Abstract

Background: The traditional home care model entails caring “for” people with disabilities, not “with” them. Reablement care has been applied to long-term care, but the evidence for care attendants, home care recipients, and family caregivers simultaneously is limited. Methods: First, a survey was conducted to explore the needs of home care recipients and family caregivers to achieve independence at home to develop the reablement home care model for home care. Then, an intervention with two groups was implemented. The experimental group included a total of 86 people who participated in the reablement home care model. The control group included 100 people and received usual home care. The self-reliance concept, job satisfaction, and sense of achievement for care attendants; quality of life for home care users; and caregiving burden for family caregivers were assessed. Results: The reablement home care model improved the job satisfaction and achievement of home care attendants, improved mutual support and independence in the self-reliance concept and quality of life among the users, and reduced the stress of the users and family caregivers. Conclusion: The reablement home care model improved the outcomes for providers, care recipients, and family caregivers. Reablement home care is suggested in long-term care policies.

## 1. Introduction

Home care (HC) aims to provide intermittent care for people with disabilities living in the community to advocate aging in place. The broad definition of HC usually includes medical, home medical, or nursing care, as well as home services that provide personal care and assistance with housework for people with disability at their homes [1]. Due to various complicated care needs and differences in home settings, HC can be both helpful and challenging. Past studies have already provided evidence that HC services can improve the physical function and quality of life of people with disabilities and reduce the burden on family caregivers [2,3]. However, HC is not a panacea. HC recipients may depend on home help too much and hesitate to do the housework that they are capable of doing by themselves [4]. In such cases, the potential of HC recipients to recover their physical function is not optimized. At times, care recipients or family caregivers might abuse HC attendants by having them perform house chores that are unrelated to the care itself and are usually performed as a system of uniform care. Person-centered care should emphasize autonomy and choice for health care recipients [5,6]. Thus, the home care that is provided to each person with a disability should be tailored to his or her own needs, preferences, and choices [7,8]. Furthermore, despite the increased expenditure associated with HC services, the independence of people with disabilities has not improved, and their quality of life worsens as their physical function declines [9]. The ability people with disabilities have to fit their home environment may be reduced due to their disabilities, and thus person–environment fit may also influence the effects of HC services. The provision of home care that fits the home environment of people with disabilities should be personalized. Therefore, a new care model called reablement care has been developed and implemented in long-term care institutions and HC settings. In this study, we aimed to establish a reablement model for HC services and to evaluate the effectiveness of this model for care attendants, care recipients, and family caregivers. The term “home care” (HC) in this study refers to personal care and housework provided for people with disabilities at home in the community and does not include medical and nursing care, and we use the term “RHC” for the reablement model of home care. In Taiwan, the requirement for people with disabilities to receive HC is a disability lasting for more than 6 months. Thus, home care is primarily provided as long-term care rather than subacute care.

The traditional HC model entails caring “for” a person with a disability and not “with” them. This care model was suggested to cause dependency and further loss of function for care recipients [4]. A new concept, i.e., reablement, aims to help people with disabilities recover their physical function through performing activities of daily living rather than having formal or informal caregivers perform these activities for them [10], focusing on supporting independence [11]. Reablement and rehabilitation are different: Reablement aims to reduce the need for long-term care by helping recipients regain confidence and learn the skills needed to maximize their independence, whereas rehabilitation aims to restore physical function to achieve the highest level [12]. Rehabilitation is a part of medical care and is often applied at the subacute care stage for persons who have a good chance of recovering completely. Although home-based rehabilitation is also available in some home care services in Taiwan, the traditional HC model used in this study did not cover home-based medical care, nursing care, or rehabilitation. In contrast, long-term care recipients with disabilities are usually unable to completely recover. In other words, rehabilitation aims to cure, but reablement aims to improve or maintain independence. Reablement often assists people with disabilities in setting their own goals to achieve independence not only by health care professionals but also by care attendants and family caregivers. Therefore, the reablement model is person-centered, preference-based care and emphasizes self-determination. The home care model in this study does not include medical care. Home care attendants can assist personal care-oriented home care for reablement.

The reablement concept is practiced at home [13]. The priority for daily activities is determined by the individuals so that timely rehabilitation and practice of daily activities can be designed and provided [14]. Lewin [15] suggests the following principles for the practice of reablement: real needs in formal care, time limit on the intervention (usually 6–12 weeks), intense intervention provided at the care recipient’s home, maximizing independence, person-centered, and goal-oriented.

This type of model has been applied for the care of people with disabilities. In the United Kingdom, Australia, and Scandinavian countries, this model has been applied in community-based care [12,16,17], whereas in New Zealand, Japan, and Taiwan, similar models, usually called self-reliance or self-support models, are applied in long-term care institutions [18,19,20]. Some empirical evidence shows that such reablement or restorative care models may improve physical function [16,18,21]. Client-centered home care may be responsive in time, safe, and improve the continuity of care [22]. By strengthening intrinsic motivation via the participant’s willpower, responsibility, and confidence, the skills needed to perform daily activities can be improved and maintained [13]. Most importantly, client-centered home care emphasizes the need for personalized customization [23]. The cost of care can be reduced by improving physical independence [24]. However, the effects of such reablement programs on care recipients’ autonomy and willpower have not been assessed. The effects of these programs on physical function or mortality are unconfirmed [17,18,25]. The effectiveness of such reablement projects on care attendants and family caregivers has only been assessed by limited research. Restorative care may improve family caregivers’ health-related quality of life [18], and family caregivers can gain more free time when reablement helps to improve the patient’s physical functions [26].

The HC model in Taiwan is primarily conducted in the traditional way. In this study, we aim to develop an RHC model for older adults with disabilities in the community through a quasi-experimental design to compare the effects of the RHC model and the traditional HC model on care attendants, care recipients, and their family caregivers.

## 2. Materials and Methods

This study consisted of two stages. First, a survey was conducted to determine the most important independence needs when designing an RHC model. Then, an experimental design was implemented to compare the effects of RHC and traditional HC on care attendants. The study gained approval from the relevant medical research ethical committees before conduction (JEN-AI IRB106-07).

### 2.1. Needs Assessment for Setting Priorities to Establish the RHC Model

We used a survey to explore participants’ needs in HC. In total, 210 care attendants, 55 HC recipients, and 65 family caregivers from two home care agencies participated in the survey. The recipients and their family caregivers were asked about their disability level and the daily activities they hoped to improve. The care attendants were asked about their needs when providing HC and rated their own professional knowledge and skills (Table 1). The most prevalent functional impairments in activities of daily living (ADLs) were taking baths, dressing, and transferring, and the most prevalent functional limitations in instrumental activities of daily living (IADLs) were heavy housework, going out by taking a car or train alone, and light housework. We also listed the expectations of the daily activities to be improved from the home care recipients and family caregivers and the most needed skills in home care from care attendants. The priority needs in home care for people with disabilities were bathing, taking a car or train alone, housework, and dressing; the priority needs in home care for family caregivers were bathing, walking, and going outside. The priority needs for care attendants to learn in home care skills were transferring, passive motion exercise, and using assistive devices. The common priorities for RHC by HC recipients, family caregivers, and care attendants were transferring and mobility as well as personal hygiene, and bathing knowledge and skills related to other care needs were provided as well. Thus, these items were set as the priority to be included in the RHC program.

The RHC program was developed by a team that was led by the researcher, who has degrees in both nursing and social work and familiarity with RHC, and consisted of a physical therapist, a nurse, a social worker, and the related staff in the home care agencies. Before the program started, the researchers communicated with the professionals about the concept of reablement and the way to deliver training for care attendants. The structure of the program to empower the care attendants was designed according to the independent care plan guide [27] (Supplemental Table S1). The training course for developing the RHC program included the concepts and skills needed for reablement in home care, and the advanced skills from the expectations based on the needs assessment survey were strengthened in the course. The main priorities for RHC were the core skills needed to empower care attendants. For example, if the most necessary skill was about transferring care, then the skill was taught by a physical therapist in the training program for care attendants. When the RHC program started, the participants (both home care recipients and their family caregivers) were asked about their special needs for their functional improvement (one or two items), and then, the care attendants developed their personalized care plan for RHC. If the care attendants needed extra mentoring regarding the care skills needed to meet the special needs of the participants, then they were able to participate in periodic meetings to consult with cross-disciplinary professionals to receive advice or further training.

### 2.2. Recruitment, Implementation, and Evaluation of the Intervention

**Figure 1:**
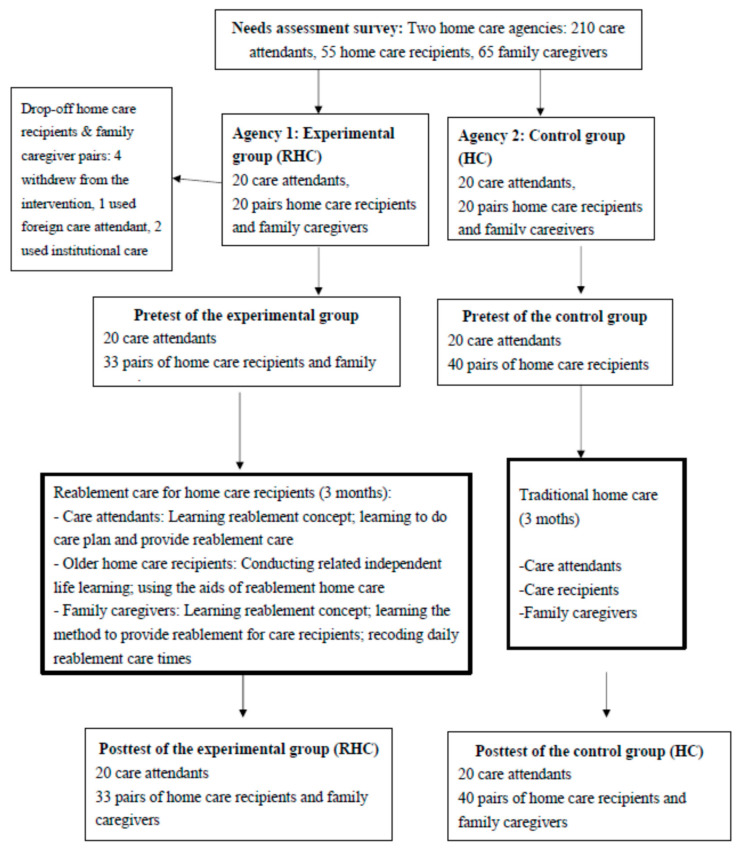
Flow chart of the intervention of the reablement home care model.

Two HC agencies participated in this survey. One home care agency was recruited as the experimental group and implemented the RHC program; the other agency was invited as the control group and implemented traditional HC service. The inclusion criteria for the participants in the two groups were older adults aged 65 years and above with a disability who were able to communicate, intact cognitive function (records from the agencies), and willing to participate in the program. The existing home care recipients and their family caregivers in these two agencies were approached and invited to participate in this study. When they understood the whole program and agreed to participate in the program, consent was provided, and the process was assessed every month during the study. The RHC group consisted of 33 pairs of care recipients and family caregivers with 20 care attendants, whereas the traditional HC (control) group consisted of 40 pairs of care recipients and family caregivers with 20 care attendants (see Figure 1). The intervention was conducted for 3 months.

The care attendants in the experimental group participated in the empowerment training program. The knowledge and skills learned by the care attendants were assessed by testing their knowledge and skills after the training and during three meetings hosted by the researcher and the care attendants during the program to solve the challenges of the RHC model.

Both the RHC recipients and their family caregivers in the experimental group were introduced to the new care model and agreed to participate in the study. In the RHC model, not only were new methods of care given to them but they also learned how to use assistive devices to improve home care recipients’ independence, and the family caregivers learned how to assist the care recipients in achieving the goal of self-reliance. The care attendants needed to work with the family caregivers to identify the most needed care items through communication, set care goals together, and help family caregivers provide RHC for the care recipients with disabilities that would encourage the care recipients’ willingness to be self-reliant and independent.

During the intervention, periodic meetings were conducted each month, not only to provide consultations with cross-disciplinary professionals to give advice or further training but also to collect qualitative observations and opinions from the care attendants during the program. The observations from the care attendants were recorded to improve the care delivery or to modify the care plan for process evaluation.

### 2.3. Measures

#### 2.3.1. Main Dependent Variable: The Self-Reliance Concept on the HC Scale

The self-reliance concept on the HC scale was based on the dynamic self-determination model [28] and modified (see Supplemental Table S2). The scale was validated by three experts in the fields of nursing, social work, and long-term care policy. The scale was tested and modified before formal application. Dependence was defined as the extent to which home attendants, home care recipients, and family caregivers depended on HC services to address their functional limitations. Mutual support was defined as the knowledge and skills that the family caregivers perceived themselves as having regarding assistance with care needs and their willingness to collaborate with the HC attendant to provide care by setting appropriate goals and learning care skills. There were 11 items in this domain for the HC attendants and care recipients and 10 items for the family caregivers. Independence was defined as the belief, willingness, and ability to help the care recipients with self-care activities to maximize their independence in HC. Each item was scored from 1 to 5. The Cronbach’s α of the scale ranged from 0.71 to 0.78, indicating satisfactory internal consistency. The development of the scale was reported previously [29].

#### 2.3.2. Variables for the Care Attendants

The HC competency test included a knowledge test of RHC and a practical skills test with scores ranging from 0 to 100. The control group was only tested on the concept of self-reliant support HC because there was no training intervention. Job satisfaction of the HC attendants included 11 items, and each item was scored from 1 to 5. The sense of accomplishment scale described the perceived achievements of the HC attendants in their job. There were 10 items on this scale, scored from 1 to 5 for each item. Demographics included gender, age, education level (elementary school or lower, junior high school, senior high school, college, university and above), marital status (having spouse or having no spouse), ethnic group, religious beliefs, work experience (years), and work hours per month.

#### 2.3.3. Variables for the Care Recipients

Disability level was measured by the Barthel Index [30], which categorizes the disability level into mild (score 91–99), moderate (score 60–90), and severe (score < 60). Cronbach’s α was 0.89, with a measurement of 10 items scored on scales of 0, 5, or 10 points. The total scores were categorized as follows: completely dependent (0~20), heavily dependent (21~61), moderately dependent (62~90), and independent (91~99). The self-care difficulty for 10 daily living activities was self-rated, including dressing and undressing, bathing, dining, taking medication, personal hygiene, transferring, bowel control, bladder control, ability to use assistive devices, and ability to perform general daily activities. Each item was rated as no difficulty, slightly difficult, very difficult, or not possible at all (scored 0 to 3); the total score ranged from 0 to 30. The WHO Quality of Life-BREF [31] was used to measure the care recipients’ health-related quality of life. There were 28 items on this scale, and each item was scored from 1 to 5. Demographics included age, gender, education, marital status, ethnic group, religious beliefs, and disability level (mild/moderate/severe).

#### 2.3.4. Variables for the Family Caregivers

The level of difficulty of caring for the care recipients was assessed for the following 10 items: dressing and undressing, bathing, eating, taking medication, personal hygiene, transferring, bowel control, bladder control, ability to use assistive devices, and ability to perform general daily activities. Each item was rated as presenting no difficulty, a slight difficulty, a great deal of difficulty, or not possible at all (scored 0 to 3). The total score ranged from 0 to 30. The family caregiving burden was measured by the 14-item Family Caregiving Burden scale [32], and each item was scored from 0 to 3. Demographics included age, gender, education, marital status, caregiving experience (years), and the relationship to the care recipients.

### 2.4. Analysis

Descriptive analysis was conducted, and the chi-square test, paired t-test, and one-way ANOVA were performed. The generalized estimation equation (GEE) [33] was used to examine the effect of the interventions by controlling for covariates. IBM SPSS 22.0 software (IBM, Armonk, NY, USA) was used for the analyses.

## 3. Results

Table 2 shows the descriptive data for the care attendants in the experimental group and the control group. The characteristics of the two groups were generally similar, except the characteristics of the care attendants at baseline.

Table 3 shows the descriptive characteristics of the care recipients and their family caregivers in the two groups at baseline. The HC users in the experimental group were younger and more likely to be male than those in the control group. The caregiving burden was higher in the experimental group at baseline.

Table 4 shows the results of pretest and post-test scores on the self-reliance HC scale. The RHC care attendants’ dependence was reduced and mutual support and independence were increased after the intervention. Both the care recipients and family caregivers reported a significant decrease in their dependence after the intervention. The RHC care attendants reported lower dependence but higher mutual support than the care recipients and family caregivers after the intervention. The self-reliance concept for the care attendants or the care recipients in the control group did not change. However, the family caregivers of the control group indicated that mutual support and independence increased over time.

Table 5 shows the results of the GEE analysis of the effects of the self-reliance HC program by comparing the experimental group and the control group. The term “group* time” indicates the effect of the self-reliance HC intervention on the experimental group compared with the effect of traditional HC on the control group. Due to the limited number of cases, only covariates that were significant in the bivariate analysis were included in the models.

After the intervention, compared with the control group, the care attendants in the experimental group who had received the self-reliance HC training had a higher training score (β = 14.66), higher job satisfaction (β = 3.35), and higher sense of achievement (β = 5.10) and rated the care recipients as having lower dependence (β = −0.505), higher mutual support (β = 11.70), and higher independence (β = 6.75) after the intervention. Regarding the care recipients, satisfaction with the HC agency increased (β = 2.79) and quality of life improved (β = 7.77). Family caregivers indicated an increased self-reliance independence level (β = 6.44) and a reduced caregiving burden (β = −2.79) after the intervention.

## 4. Discussion

This quasi-experimental study established a new RHC model and evaluated its effects on care attendants, care recipients, and family caregivers. The intervention was found to be beneficial for the care attendants’ perception of their self-reliance HC skills, job satisfaction, and sense of achievement at work and led to higher mutual support and independence among their care recipients. The RHC recipients indicated that the new care model improved their quality of life and reported higher satisfaction with the HC provider. The RHC model increased the perceived independence of the family caregivers and reduced their caregiving burden.

### 4.1. Effects for Home Care Recipients

The reablement intervention was shown to improve quality of life; although the difficulty with self-care did not change significantly, in contrast with the improvement in physical function reported in previous studies [16,19,21]. However, the intervention helped to reduce the care recipients’ perceived dependence and increase mutual support, although the change was not significant. Because the disability level is often unable to be recovered completely, maintaining current physical function for as long as possible should be the goal. In addition, it is possible that physical function was not sensitive enough to such a short-term intervention and required more time to change participants’ mindset and behaviors from mutual support to independence. In this study, the duration of the program was set to 3 months according to the existing literature [15]. The item number for developing care plans in RHC was set to 1 or 2. Under this short-term period and with limited items for functional reablement, it was easier and practical for home care recipients and care attendants to perform such an RHC care plan.

### 4.2. Effects for Family Caregivers

Previous research has indicated that reablement care may improve family caregivers’ quality of life [18] and provide some relief from care [26]. In the current study, the RHC model reduced the caregiving burden and significantly increased the independence concept in caregiving. The involvement and responsibility of the care recipients in the process made them willing to try, and at best, their function could be improved. The change in the mutual support and independence of people with disabilities could help family caregivers feel supported as part of a team with the care recipient and the care attendant. Such support would mean that family caregivers would not need to feel responsible for caring for the person with a disability alone. The increase in the independence concept of family caregivers may also change their expectations of care and ways to care for adults with disabilities. Traditional culture in Taiwan expects family members to care for older adults with disabilities to show their filial piety or ask care attendants to do most of the work, even though adults with disabilities may still have some degree of function. The reablement concept of family caregivers would help them build appropriate expectations in home care and encourage adults with disabilities to improve their physical function as much as possible.

### 4.3. Effects for Care Attendants

The self-reliance concept of the reablement program affected care attendants by reducing their expectations of care recipients’ dependence while increasing their emphasis on mutual support and independence through HC. The care attendants in the experimental group increased their competency, job satisfaction, and sense of achievement after the intervention. Care attendants are often viewed as semiprofessionals, and their sense of achievement and dignity have usually been neglected. Through the empowerment process, the reablement process gives care attendants more autonomy and a greater sense of achievement in their work [34].

### 4.4. Process Evaluation

Reablement constitutes a paradigm shift in community medical services from a traditional model that focuses on alternative functions to a supplemented a model centered on activities and independence [35]. The development of the individualized RHC program was challenging for the HC attendants, despite support and assistance from the researchers and professionals in the HC agency. According to the opinions collected during the program, the RHC care attendants reported that it was hard to change the HC method at the beginning. They found that using an authoritative attitude to require care recipients’ cooperation did not help. In contrast, if they acted like a partner and asked the care recipient to be their partner in the reablement activities, the effect was much better. Family caregivers’ attitudes and cooperation were very important to assure the efficacy of the program when the care attendants were not there. Communication skills, such as empathy, listening, and the ability to let the participant be an expert in his or her life situation, are prerequisites for allowing the participants to set their own goals [36]. Some of the care recipients reported that “they feel alive” because they were doing something for themselves, similar to previous findings [26]. Reablement care also increased the interaction between the care recipients and both the care attendants and family caregivers. Trust in the care attendants and the relationship with family caregivers were built through the reablement process.

### 4.5. Strengths and Limitations

This study has strengths. To the best of our knowledge, this is the first reablement intervention program to consider the effect not only on care recipients but also on caregivers, both formal care attendants and family caregivers. The reablement concept was proven to be feasible in the HC setting and not only in institutions.

This study has limitations. First, the study was conducted in only two HC agencies. The results may not be generalized to other agencies, care recipients, or family caregivers. Second, the sample size was not large, and the study was not a randomized trial due to limitations. It was not possible to use a double-blind design for this study. Third, more time may be needed to show more significant effects of RHC. Fourth, it was not feasible to conduct a randomization intervention because we needed to obtain agreement from the home care agency and the care recipients. Thus, only a quasi-experimental design was conducted. In addition, the competency of care attendants of the RHC program was better than that of the control group, and their working tenure was also longer than that of the control group. RHC care attendants may be more capable of delivering advanced home care skills at baseline. Thus, an advanced statistics method (GEE) was applied. The group difference was controlled in the GEE analysis, and the intervention effect (the time*group term) was still significant, which means that the RHC program did show a significant effect when controlling for group (agency) differences. Fifth, the participants who agreed to participate in the RHC program may have had higher motivation to improve their physical function, and thus the effect for them may have also been better. There may have been a self-selection bias in the participants. Sixth, the study focused on the quantitative outcomes of the program, and only limited qualitative findings were observed in the process. A mixed methodology for evaluating the program is suggested in future research.

## 5. Conclusions

This study provides evidence regarding the establishment and evaluation of an RHC model for older adults with disabilities. We suggest that additional research on the reablement care model should be developed to further support its effects. If additional evidence in support of reablement care is accumulated, then the RHC model could be incentivized in the payment system to encourage the provision of HC quality in long-term care policy.

## Figures and Tables

**Table 1:** Needs assessment of the reablement home care items reported by care recipients, family caregivers, and care attendants.

Care Recipients (*n* = 55)		Family Caregivers (*n* = 65)		Needs of Care Attendants (*n* = 210)	
Variables	Mean (SD) or %	Variables	Mean (SD) or %	Variables	Mean (SD) or %
Age	79.7 (8.9)	Age	59.2 (11.1)	Age	53.2 (7.2)
Sex		Sex		Sex	
Male	63.6%	Male	32.3%	Male	5.2%
Female	36.4%	Female	67.7%	Female	94.8%
Years of home care use		Years of family caregiving		Working years in home care	
≤1 year	30.9%	≤1 year	18.5%	<3 years	22.9%
2–3 years	50.9%	2–3 years	29.2%	4–5 years	28.6%
4–5 years	14.5%	4–5 years	13.8%	6–10 years	34.3%
6 or more years	3.6%	6 years or more	38.5%	More than 10 years	14.3%
Care needed in ADLs		Relationship with home care recipients		Expectation for advanced knowledge	
Taking a bath	72.7%	Spouse	41.8%	Personal care	4.8%
Dressing	54.5%	Sons or daughters	40.0%	Housework	26.7%
Eating	12.7%	Daughters-in-law	7.3%	Preparing food, shopping, etc.	1.5%
Transferring	34.5%	Others	10.9%	Emergency	2.4%
Using toilet	16.4%	Home care use items		End of care	22.8%
Walking indoors	27.3%	Personal care (ADLs)	83.1%	Stress coping	12.9%
Care needed in IADLs		Housework	23.1%	Occupation injury prevention	16.6%
Shopping for groceries	67.2%	Preparing meals, accompany or shopping groceries	41.5%	Infection prevention	16.7%
Managing money	47.3%	Accompany for rehab	16.9%	Resource connection	23.8%
Taking train/car alone	83.6%	Others	13.8%	Dietary nutrition	12.4%
Heavy housework	89.1%	Expectation of from home care in ADLs		Expectation to learn professional skills (top 5)	
Light housework	78.2%	Eating	15.4%	Transferring	33.9%
Making phone calls	47.3%	Grooming	16.9%	Passive Range or Motion Exercise	26.7%
Expectation to improve in ADLs		Using toilet	15.4%	Using assistive devices	26.2%
Taking a bath	50.9%	Taking a bath	55.4%	Emergency and CPR	15.7%
Dressing	41.8%	Walking	35.4%	Baths and body cleansing	13.3%
Eating	16.4%	Going up/down stairs	24.6%		
Transferring	27.3%	Dressing	21.5%		
Using toilet	21.8%	Bowel control	7.7%		
Walking indoors	27.3%	Bladder control	3.1%		
Expectation to improve in IADLS		Expectation of from home care in IADLs			
Shopping groceries	25.5%	Shopping groceries	20.0%		
Managing money	3.6%	Going out	41.5%		
Taking train/car alone	27.3%	Preparing food and cooking	38.5%		
Heavy housework	12.7%	Housework	33.8%		
Light housework	40.0%	Laundry	16.9%		
Making phone calls	5.5%	Making phone calls	1.5%		
		Taking medicine	13.8%		
		Managing money	1.5%		

ADLs: activities of daily living; IADLs: instrumental activities of daily living.

**Table 2:** Description of the sample: care attendants.

Variables	Experimental Group (*n* = 20)	Control Group (*n* = 20)	Significance (Two Groups Baseline)
Age	51.50 (5.93)	55.20 (7.80)	
Gender			
Male	10.0%	15.0%	
Female	90.0%	85.0%	
Education			
Elementary school or lower	0.0%	15.0%	
Junior high school	15.0%	20.0%	
Senior high school	65.0%	50.0%	
College/university or above	20.0%	15.0%	
Marital status			
Having spouse	75.0%	65.0%	
No spouse	25.0%	35.0%	
Working experience			
≤3 years	25.0%	15.0%	
4–5 years	40.0%	30.0%	
6–10 years	25.0%	30.0%	
≥11 years	10.0%	25.0%	
Working hours per month	173.50 (33.95)	146.35 (22.94)	**
Competency test (pretest)	78.72 (3.63)	70.30 (4.28)	***
Competency test (posttest)	93.90 (4.58)	70.79 (3.59)	
Sig. (pre-post)	***		
Job satisfaction (pretest)	39.65 (4.40)	43.75 (3.31)	**
Job satisfaction (posttest)	44.30 (5.07)	45.05 (3.59)	---
Sig. (pre-post)	**	*	
Sense of accomplishment for work (pretest)	36.90 (3.19)	37.55 (3.95)	
Sense of accomplishment for work (posttest)	43.45 (3.97)	38.80 (4.18)	---
Sig. (pre-post)	***	*	

Analysis by the *t* test or Chi-square test. Significance was compared across two groups at baseline (shown in the last column) and the pre-and post-tests (shown within the group column). * *p* < 0.05, ** *p* < 0.01, *** *p* < 0.001.

**Table 3:** Description of the sample: home care users and family caregivers.

Home Care Recipients at Baseline			Family Caregivers at Baseline		
Variables	Experimental Group (*n* = 33)	Control Group (*n* = 40)	Variables	Experimental Group (*n* = 33)	Control Group (*n* = 40)
Age	74.27 (10.1) *	79.32 (8.0)	Age	57.39 (13.4)	55.38 (12.9)
Gender	**		Gender		
Male	57.6%	25.0%	Male	39.4%	50.0%
Female	42.4%	75.0%	Female	60.6%	50.0%
Education			Education		
Elementary school or lower	51.5%	55.0%	Elementary school or lower	30.3%	15.0%
Junior high school	6.1%	15.0%	Junior high school	6.1%	25.0%
Senior high school	21.2%	12.5%	Senior high school	24.2%	35.0%
College/university or above	15.1%	2.5%	College/university or above	33.3%	25%
Others	6.1%	15.0%	Others	6.1%	0%
Marital status			Marital status		
Having spouse	48.5%	30.0%	Having spouse	72.7%	67.5%
No spouse	51.5%	70%	No spouse	27.3%	32.5%
Disability level			Caregiving experience		
Mild	24.2%	25.0%	≤1 year	6.1%	7.5%
Moderate	39.4%	32.5%	2–3 years	27.3%	37.5%
Severe	36.4%	42.5%	4–5 years	9.1%	7.5%
Self-care difficulty (pretest)	12.45 (8.74)	14.95 (9.67)	6 years or more	57.6%	47.5%
Self-care difficulty (posttest)	11.88 (8.77)	13.00 (9.20)	Relationship		
Sig. (pre-post)		*	Spouse	30.3%	20%
Quality of life (pretest)	76.5 (12.2)	76.4 (11.6)	Children or daughter-in-law	45.5%	72.5%
Quality of life (posttest)	84.61 (6.1)	76.68 (11.4)	Brothers or sisters	9.1%	5.0%
Sig. (pre-post)	**		Others	15.1%	2.5%
			Work		
			Yes	36.4%	42.5%
			No	63.6%	57.%
			Caregiving burden (pretest)	24.94 (7.6) *	20.30 (8.5)
			Caregiving burden (posttest)	23.6 (7.7)	21.7 (8.9)
			Sig. (pre-post)		*

Analysis by the *t* test or Chi-square test. * *p* < 0.05, ** *p* < 0.01.

**Table 4:** Pretest and post-test of the self-reliance home care scale rated by the care attendants, home care recipients, and family caregivers.

	Experimental Group				Control Group			
Pretest	Care Attendants	Care Recipients	Family Caregivers	Significance	Care Attendants	Care Recipients	Family Caregivers	Significance
Dependence	25.55 (1.57)	27.06 (7.08)	25.58 (3.20)		23.05 (2.65)	24.83 (3.05)	24.65 (2.98)	
Mutual-support	43.35 (2.29)	45.03 (4.36)	41.21 (5.44)	**	47.95 (5.99)	46.15 (6.01)	42.65 (4.73)	**
Independence	42.05 (1.82)	44.27 (5.45)	43.94 (6.32)		41.05 (4.29)	44.13 (7.71)	45.50 (5.40)	*
Posttest	Care attendants	Care recipients	Family caregivers	Significance	Care attendants	Care recipients	Family caregivers	Significance
Dependence	20.35 (2.01) ***	24.45 (2.69) *	24.21 (3.10) *	***	22.90 (2.38)	24.48 (3.08)	24.08 (2.91)	
Mutual-support	52.25 (3.46) **	46.06 (4.88)	42.18 (4.68)	***	47.95 (5.10)	44.75 (4.91)	41.25 (3.64) *	***
Independence	48.35 (4.09) **	44.97 (6.55)	44.76 (5.15)		40.60 (4.03)	45.03 (6.70)	39.88 (4.13) ***	**

Analysis by one-way ANOVA (among two groups of participants, significance is shown in the last column) and the paired *t* test (within participants pre- and posttests, significance is shown in the posttest cell). * *p* < 0.05, ** *p* < 0.01, *** *p* < 0.001.

**Table 5:** Effects of self-reliance support intervention on the care attendants, care recipients, and family caregivers by the generalized estimating equation.

**Care Attendants**	**Competency Test**	**Job Satisfaction**	**Sense of Achievement**	**Dependence**	**Mutual Support**	**Independence**	
Seniority	−0.41	0.94	0.79	−0.25	0.31	0.78	
Group	8.23 ***	−3.68 **	−0.10	2.39 ***	−4.46 **	1.35	
time	0.53	1.30 **	1.25 **	−0.15	−2.80 **	−0.45	
Group * time	14.66 ***	3.35 ***	5.10 ***	−5.05 ***	11.70 ***	6.75 ***	
AIC	1878.26	1255.32	1102.86	369.50	1215.00	1005.68	
**Home care recipients**	**Self-care difficulty**	**Satisfaction to home care**	**Satisfaction to home care agency**	**Dependence**	**Mutual support**	**Independence**	**Quality of life**
Degree of disability	7.32 ***	4.91 **	0.95	0.36	0.75	0.54	−0.24
Group	−2.10	2.46	−2.18	2.23	−1.08	0.18	0.15
time	−1.95 *	2.20	−1.00	−0.35	−8.933 × 10^−17^	0.90	0.33
Group * time	1.37	−0.14	2.79 *	−2.23	1.03	−0.20	7.77 **
AIC	7013.34	25,808.17	5767.84	2568.99	3751.61	6386.99	16,358.89
**Family caregivers**	**Care difficulty**	**Satisfaction with home care**	**Satisfaction to home care agency**	**Dependence**	**Mutual support**	**Independence**	**Caregiving burden**
Degree of disability	1.24	−3.94 **	−1.91 *	-.32	−1.80 *	−1.61	2.68
Having a job	−0.16	−1.80	−1.43	−1.10	−2.15 *	−1.74	−2.86
Group	−1.76	−0.42	−1.02	0.84	−1.64	−1.73	4.56 *
time	−1.05	0.65	0.20	−0.58	−1.40*	−5.63 ***	1.40 *
Group * time	0.32	−0.68	−0.05	−0.79	2.37	6.44 ***	−2.79 ***
AIC	7100.51	6508.59	2506.03	1283.40	2886.93	3831.04	8925.97

The control group was the reference group. AIC stands for Akaike Information Criterion. * *p* < 0.05, ** *p* < 0.01, *** *p* < 0.001.

## Supplementary Materials

The following are available online at https://www.mdpi.com/1660-4601/17/23/8784/s1, Table S1. The content of the reablement home care model for empowering the care attendants; Table S2. The self-reliance home care scale.
