# Supplementary material for: Evaluation of Reablement Home Care: Effects on Care Attendants, Care Recipients, and Family Caregivers
Source: Int J Environ Res Public Health. 2020 Nov 26;17(23):8784. doi: 10.3390/ijerph17238784 (PMC7730042; doi:10.3390/ijerph17238784)
Supplement: Supplementary file 1 [file ijerph-17-08784-s001.pdf]

International Journal of
Environmental Research and Public Health

MDPI

Supplement Table S1. The content of the reablement home care model for empowering the care attendants

Topics	Contents
Concept of reablement and self-reliance	Experiencing the difficulty of disability, concept of reablement, comparison of traditional care and self-reliance home care, care knowledge basis
Personal hygiene skills	Bathing/showering, dressing and changing clothes, using toilet and cleansing,
Transferring and mobility skills	Skills in turning over, care for sitting up, balance of sitting, care for standing up, balance of standing, care for walking, care for climbing stairs, transferring (bed-chair-wheelchair)
Use of assistive devices	personal care assistive device, transferring and mobility assistive devices, personal care and protective assistive devices, fall preventing assistive devices, resource connection of assistive device
Home environment assessment	home care setting assessment and modification for barrier-free environment,
Home rehabilitation activities	Knowledge of rehabilitation, joint activities, limb recession prevention, rehabilitative activities for long-term bedded users, activity design
Individualized home care plan	Physical function in activities of daily living and instrumental activities of daily living, scale assessment skills and goal setting, communication skills, use of sympathy

Supplement Table S2. The self-reliance home care scale

**Dependence in home care**
1.The longer hours of home care services, the more help I get.
2. The more items of home care I use, the more assistance I get.
3. The goal of home care is to get the daily activities (such as taking a bath, housework) that I can't do done for me.
4. Home care can replace all the self-care function.
5. When the family caregiver provides care, they usually don't know the caring skills and depend on home care attendants.
6. I can feel the caregiving pressure perceived by the family caregivers.
7. The home care service from the agency meets my needs.
**Mutual-support in home care**
8. The home care attendant usually discuss the service content or method of care with me.
9. The home care attendant provide some methods to help me realize the self-care skills.
10. I am willing to let care attendants teaching me how to accomplish self-care (such as taking a bath, walking, etc.)
11. Home care can reduce my family caregivers reducing their burden.
12. I am willing to spend time learning how to do daily activities independently.
13. I am willing to set up some goal to improve self-care ability.
14. I need assistive device (such as wheels or walker) to help me do my daily activities by myself.
15. I expect the home care attendants share the caregiving stress with family caregivers. #
16. I try my best to accomplish the daily activities by myself.
17 The home care attendants is competent in providing care to me.
**Independence in home care**
18. A good care is to help me become able to do self-care independently.
19. I am willing to modify my home setting to increase my ability in daily activities
20. I believe that I still have some ability to accomplish self-care.
21. I expect to learn self-care to reduce family caregiver's burden.
22. Family can obtain the caregiving skills and knowledge from home care services.
23. I have autonomy in deciding the care of my daily activities (such as the way to taking a shower).
24. When I can learn self-care and keep better, I can stay at home longer (from using institutional care).
25. Home care services help me live in my home comfortably and thus I don't need to worry that I have to go to a long-term care institution someday.
26. I feel the sense of accomplishment when I learn how to do self-care.
27. When I learn self-care, my family can help me in a more efficient way.
28. believe that home care is better than other ways of long-term care (e.g. day care or institutional care)
